# The therapeutic effect of the neuropeptide hormone somatostatin on *Schistosoma mansoni *caused liver fibrosis

**DOI:** 10.1186/1471-2334-5-45

**Published:** 2005-06-10

**Authors:** Shyama Chatterjee, Gunther Vrolix, Inge Depoortere, Theo Peeters, Eric Van Marck

**Affiliations:** 1 Laboratory of Pathology, Faculty of Medicine, University of Antwerp, Universiteitsplein-1, B-2610 Antwerp, Belgium; 2 Laboratory of Gastrointestinal Hormones, Gasthuisberg, Leuven, Belgium

## Abstract

**Background:**

The neuropeptide somatostatin is one of the major regulatory peptides in the central nervous system and the digestive tract. Our recent work has delineated an association between fibrosis and low levels of endogenous somatostatin plasma levels in *Schistosoma mansoni *infected subjects. Based on these results this paper explores the therapeutic potential of somatostatin in a mouse model of hepatic fibrosis associated with *S. mansoni *infections.

**Methods:**

Groups of outbred Swiss mice were infected with 100 *S. mansoni *cercariae, infection maintained till weeks 10 or 14, and then somatostatin therapy delivered in two regimens – Either a one or a two-day treatment. All animals were sacrificed one week after therapy and controlled for liver, spleen and total body weight. Circulating somatostatin levels in mice plasma were measured at the time of sacrifice by means of a radio-immuno assay. GraphPad Prism^® ^was used for statistical calculations.

**Results:**

Somatostatin administration showed little toxicity, probably due to its short half-life. Total liver and spleen weights of *S. mansoni *infected animals increased over time, with no changes observed due to somatostatin therapy. Total body weights were decreased after infection but were not affected by somatostatin therapy. Snap frozen liver sections were stained with haematoxylin-eosin or Masson's trichrome to study parasite count, hepatocyte status, granuloma size and cellularity. After somatostatin treatment mean egg counts per liver section (43.76 ± 3.56) were significantly reduced as compared to the egg counts in untreated mice after 10 weeks of infection (56.01 ± 3.34) (*P *= 0.03). Similar significant reduction in parasite egg counts were also observed after somatostatin treatment at 14 weeks of infection (56.62 ± 3.02) as compared to untreated animals (69.82 ± 2.77)(*P *= 0.006). Fibrosis was assessed from the spectrophotometric determination of tissue hydroxyproline. Infection with *S. mansoni *caused increased hydroxyproline levels (9.37 ± 0.63 μmol at wk10; 9.65 ± 0.96 μmol at wk14) as compared to uninfected animals (1.06 ± 0.10 μmol). This significant increase in collagen content (*P *= 0.01; 0.007 respectively) marks the fibrosis observed at these time points. Treatment with somatostatin resulted in a significant decrease in hydroxyproline levels both at wk10 (4.76 ± 0.58 μmol) and at wk14 (5.8 ± 1.13 μmol) (*P *= 0.01; 0.03 respectively). Endogenous somatostatin levels were increased at wk10 (297 ± 37.24 pg/ml) and wk14 (206 ± 13.30 pg/ml) of infection as compared to uninfected mice (119 ± 11.99 pg/ml) (*P *= 0.01; 0.008 respectively). Circulating somatostatin levels in infected animals were not significantly affected by somatostatin treatment. Hepatocyte status remained unaltered and granulomas were not remarkably changed in size or cellularity.

**Conclusion:**

Our experiments reveal an antifibrotic effect of somatostatin in schistosomiasis. We have previously shown that the somatostatin receptors SSTR2 and SSTR3 are present on the parasite egg and worms. We therefore hypothesize that somatostatin reduces either the number of parasite eggs or the secretion of fibrosis inducing-mediators. Our data suggest somatostatin may have therapeutic potential in *S. mansoni *mediated liver pathology.

## Background

Somatostatin (SOM) or somatostatin-14, a 14 amino acid peptide hormone was originally isolated from the hypothalamus. It was soon after reported in the central nervous system (CNS), the stomach, gastrointestinal (GI) tract and pancreas. By 1980, the gene coding for prosomatostatin had been identified and two molecular forms (SOM-14 & SOM-28) were reported. As the five specific receptors for somatostatin (SSTR1-SSTR5) belonging to the G protein-coupled receptor family were identified, it became clear that the different molecular forms and various receptors are part of a complicated control system that regulate a variety of body functions. Somatostatin exerts profound inhibitory functions not only on growth hormone secretion but also on many GI functions. These different aspects form the basis for the therapeutic potential of somatostatin in various diseases [1].

Somatostatin alone or in combination with endotherapy has been successfully used in the management of bleeding esophageal varices (BOV) [2]. Following an acute bleeding episode somatostatin is administered over a period of 5 days and acts by reducing the splanchnic blood flow. Somatostatin is useful in reducing peptide ulcer bleeding and the lack of recurrence [3]. More recently, somatostatin has also shown proven benefits in the management of pancreatic disorders, such as pancreatic fistulae, acute pancreatitis and the prevention of complications following pancreatic surgery [4-6]. Somatostatin is well tolerated, with very few side effects.

Complications resulting from hepatic fibrosis are the principal cause of death in *Schistosoma mansoni *infected patients [7]. In such patients portal hypertension leads to the formation of gastro-esophageal collaterals (varices). Fatal bleeding of these esophageal varices can occur depending on the severity of fibrosis. We have previously shown an association between severe hepatic fibrosis and low levels of endogenous somatostatin [1]. Taking these reports into consideration, our present study was aimed to test the therapeutic capacity of exogenously administered somatostatin on *S. mansoni *caused liver pathology.

In schistosomiasis the principal inflammatory response is directed against the parasite eggs some of which enter the portal circulation, become lodged in hepatic portal venules initiating a granulomatous response. During periovular granuloma formation fibronectin produced by macrophages is deposited around the inflammatory cells [8,9] composing a large part of the extracellular matrix. This is followed by the deposition of the proteoglycans dermatan sulphate, to a lesser extent heparan sulphate [10], and the interstitial collagen types I & III that all together form the fibrotic tissue.

**Figure 1:**
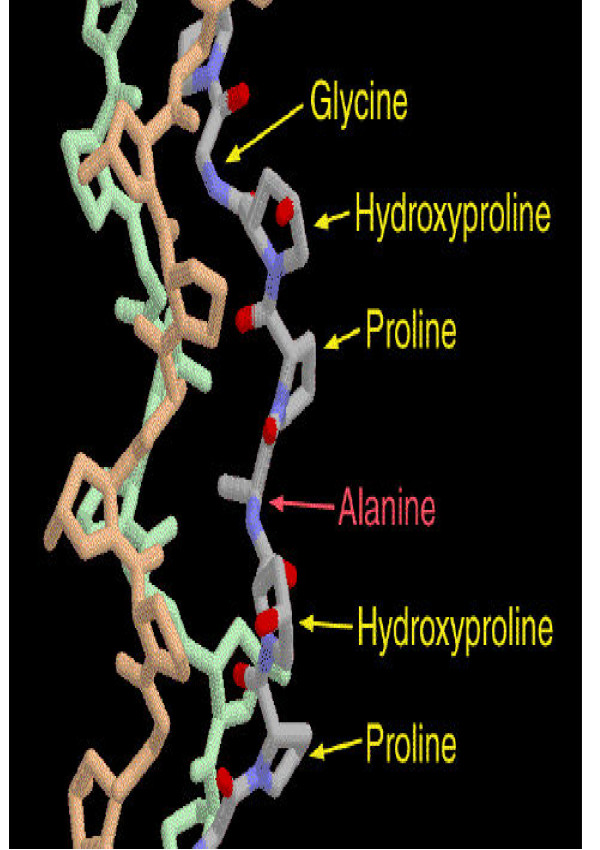
Structure of collagen triple helix.

Collagen is composed of three chains, wound together in a tight triple helix (Fig. 1.). A repeated sequence of three amino acids forms this sturdy structure. Every third amino acid is glycine, a small amino acid that fits perfectly inside the helix. Many of the remaining positions in the chain are filled by two unexpected amino acids: proline and a modified version of proline, hydroxyproline. The glycine forms a tiny elbow packed inside the helix, and the proline and hydroxyproline smoothly bend the chain back around the helix.

Our recent studies have elucidated the presence of somatostatin receptors (SSTR2A, mostly associated with inflammatory cells, and receptor SSTR3 expressed in the liver) on *S. mansoni *egg and worm stages (manuscript submitted). Soluble egg antigen (SEA) secreted by the egg stage parasite in the liver triggers inflammation and fibrosis [11]. Circulating host somatostatin levels might inhibit SEA production via interaction with the SSTR receptors on the parasite surface, thereby regulating the level of liver fibrosis and as a result portal hypertension, variceal bleeding and fatality due to this disease. In related studies the group of Reynaert [12] have shown that the presence of SSTRs 1, 2 and 3 on hepatic stellate cells could be used as therapeutic targets for reducing fibrosis in chronic liver disease because treatment with somatostatin decreased collagen I and III and alpha-SMA synthesis.

Based on these reports we presented the hypothesis that exogenous administration of somatostatin could alleviate the pathology caused by schistosomiasis. To prove this we tested the effect of somatostatin treatment on collagen deposition in the liver of *Schistosoma mansoni *infected Swiss mice as compared to control uninfected or untreated mice. We have on several occasions stressed the therapeutic potential of somatostatin on *S. mansoni *caused liver fibrosis [13]. Our results may indicate an endogenous function of the somatostatin receptors on the parasite surface and point to their role in host-parasite interactions.

## Methods

### Pharmacokinetic profile of somatostatin

Separate groups of male Swiss mice were injected with 25 μg of somatostatin (somatostatin-ucb^®^, UCB Pharma, Brussels) intraperitoneally (in the abdomen) or intravenously (in the caudal vein in the tail). At defined moments after injection (10, 20, 40, 60 minutes) the animals were anaesthetized with Nembutal^® ^(60 mg/kg). The thoracic cavity was cut open and blood collected from the right ventricle of the heart into chilled syringes containing EDTA (1 mg/ml) and Aprotinin (500 KIU/ml blood). The collected blood was centrifuged at 3000 rpm for 15 minutes at 0°C. The plasma was immediately frozen at -80°C freezer. Untreated naïve mice were also bled to ascertain background levels of somatostatin.

### Schistosoma mansoni infection

The maintenance of the *S. mansoni *life cycle and the transcutaneous infection of mice with *S. mansoni *have been previously described [14]. Male Swiss mice (age 7 weeks) were anaesthetized with Nembutal^® ^(60 mg/kg) and the abdomen was shaved. A metal ring was placed on the abdomen and then filled with treated water containing infectious cercariae of a Puerto Rican strain of *S. mansoni*. The cercariae were allowed to penetrate during 20 minutes after which the water was removed and checked for remaining cercariae.

### Experimental setup

Groups of Swiss mice were infected with 100 *S. mansoni *cercariae each as mentioned above. Age matched mice were maintained as uninfected control animals. Groups of 10 mice composed Group 1 that were maintained till 10 weeks following infection, other 10 mice composed Group 2 that were maintained till 14 weeks of infection.

At such times animals of Groups 1 and 2 were treated with somatostatin (Somatostatine-ucb^®^), that was kindly gifted by UCB Pharma, Brussels. Somatostatin was administered in two regimens – a one-day treatment or a two-day treatment. One day treatment consisted of 3 doses of 30 μg somatostatin each, two day treatment was the double of this dose, that is 6 doses of 30 μg somatostatin to each animal. Somatostatin was administered intravenously via the caudal vein in the tail and intraperitoneally at regular intervals during the day. One week after the last somatostatin administration, mice were killed, the weight of the animal, liver and spleen were noted, plasma was extracted and stored as mentioned above, and the liver frozen immediately in liquid nitrogen and then at -80°C. The overall planning is depicted in Table 1. Untreated animals of the acute and chronic stage were also sacrificed at the respective times together with their treated counterparts. Research protocols involving rodents received ethical clearance by the University of Antwerp ethical committee.

**Table 1:** The experimental planning of our study is depicted.

Criteria	**Gp 1**	**Gp 1**	**Gp 1**	**Gp 2**	**Gp 2**	**Gp 2**
Time of SOM treatment	Acute (wk. 10) 1 day treatment	Acute (wk. 10) 2 days treatment	Acute (wk. 10) Untreated	Chronic (wk. 14) 1 day treatment	Chronic (wk. 14) 2 days treatment	Chronic (wk. 14) Untreated
No. doses	3 × 30 μg (2 × IP; 1 × IV)	6 × 30 μg (4 × IP; 2 × IV)	NA	3 × 30 μg (2 × IP; 1 × IV)	6 × 30 μg (4 × IP; 2 × IV)	NA
Total SOM	90 μg	180 μg	NA	90 μg	180 μg	NA

NA: not applicable; SOM: somatostatin^®^; Wk.: week; IP: Intraperitoneal; IV: Intravenous; No.: number.

### Parasite egg count and hepatocyte status after somatostatin treatment

All animals of Groups I and II were controlled for liver, spleen and total body weight, before sacrifice. The adult worms were recovered from the hepatic portal system and the liver by perfusion with citrate saline (0.85% sodium chloride; 1.5% sodium citrate). The livers were cut out and snap frozen in liquid nitrogen. For cyosectioning, liver fragments were embedded in Tissue-Tek OCT compound, 4 μm thick transverse sections were cut on a cryostat, mounted on slides coated with 0.1% ploy-L-lysine and stored at -20°C until use. To study parasite egg count, hepatocyte status, granuloma size and cellularity, series of sections were stained with Haematoxylin-eosin stain.

### Hydroxyproline determination

The collagen concentration was determined by assessing hydroxyproline amount. Herein is described the protocol of Technique B for the biochemical assessment of fibrosis used by Bergman and Loxley [15]. Just as was done by Cheever [16] we neutralized our samples for the color reaction.

#### Reagents used

1. Dowex/Norit: Twenty mg of Dowex (Sigma, St. Louis, Missouri, US) and 10 mg Norit A (Merck, Darmstadt, Germany) were mixed together in 200 ml of 6N HCl. The Dowex/Norit mixture was recovered with the help of a vacuum pump and thereafter washed thrice more with 6N HCl. The residue was rewashed with 95% ethanol and 100 % ethanol, and the powder dried over several days.

2. Solution A: One part 7% chloramine T-solution (Sigma) was mixed with 4 parts of citrate/acetate buffer (pH 6.0: 57 g sodium acetate.3H_2_O (Merck) + 37.5 g Na3citrate.2H_2_O (Sigma) + 5.03 g Citrate + 385 ml isopropanol made up to 1 liter with distilled water).

3. Solution B: Composed of 3 parts of Ehrlichs reagent (25 g p-dimethyl amino benzal dehyde (Sigma) dissolved in 37.5 ml 60% perchloric acid) and 13 parts isopropanol.

4. Hydroxyproline standard solution: 6.56 mg hydroxyproline standard (Merck) dissolved in 500 ml gave a 0.1 mM hydroxyproline-solution. This solution remains stable at 4°C thereby enabling us to set up a standard curve of known hydroxyproline values.

#### Hydrolysis of liver

For the measurement of the hydroxyproline content in the liver, about 200 mg of liver was treated with 5 ml of 6N HCl for 18 hours at 110°C. This acidic hydrolysis breaks down the collagen to individual amino acids. Remaining undissolved matter was removed by adding 40 mg Dowex/Norit in 5 ml of distilled water. After centrifugation for 15 minutes at 2000 rpm, the supernatant was filtered with the aid of 0.22 μm millipore filters (Millipore S.A., Molsheim, France).

#### Neutralization

Two ml of hydrolysate was pipetted out to which was added 1 drop (40 μl) of 1% phenolphthalein. When the solution became colorless, 10 N NaOH was added drop wise till the color changed to purple red. Return titration was done with 5 μl drops of a 3N HCl solution, till all red color was lost. The total volume was next restored to 4 ml with distilled water and the solution kept stable at 4°C.

#### Color reaction

Starting from this step we used a series of standard hydroxyproline concentrations made from 0-25-50-75-100 μmol/l. (200 μl/test tube). From the test sample above 200 μl was placed in a separate test tube. After vortexing 200 μl test sample/200 μl standard mixed together with 400 μl of isopropanol, 200 μl of solution A (chloramine T/citrate-acetate buffer) was added that provided an optimal binding between color and tissue. This reaction needed at least 4 minutes to work after which 2.5 ml of solution B was added and the contents well mixed. The tubes were covered with aluminum foil and incubated for 25 minutes in a warm water bath maintained at 60°C. To stop the reaction the test tubes were cooled in cold water for 3 minutes.

#### Measurement

Within 30 minutes, the absorbance for each sample was measured in an Ultrospec 3000 UV/Visible Spectrofotometer at a wavelength of 558 nm.

### Measurement of somatostatin levels in plasma

The measurement of somatostatin concentrations in the Swiss mice plasma was carried out in the laboratory of Gastro-intestinal Hormones, at Gasthuisberg, K.U. Leuven, by means of a radio-immuno assay (RIA). The RIA was performed by incubating the samples with 1.7 pM 3-[^125^I] iodotyrosyl^11 ^somatostatin-14 (specific activity 2000 Ci/mmol, Amersham Pharmacia Biotech, Buckinghamshire, UK) and a rabbit antibody against human SOM [1-14] in a 50 mM sodium phosphate buffer (pH 7.4, 0.25% EDTA, 0.5% charcoal-BSA, 500 U/ml Trasylol) for at least two days at 4°C. At the end of the incubation period the SOM bound to the antibody was separated from the free SOM by adding 500 μl dextran-charcoal followed by centrifugation for 15 min at 3000 rpm. Both fractions were counted in a gamma counter and the results were read from a standard curve (0–250 pg/ml) included in the RIA. The minimal detectable dose was 2.5 pg/ml.

## Results

### Toxicity

Somatostatin is a highly purified compound reflected by the fact that it has very little toxicity. Studies in mice have shown that the LD50 is comparable to 10,000 times the acute therapeutic dose used in humans. The favorable metabolic profile of somatostatin is further supported by the fact that this compound has a very short half-life, and thus any undesirable effects may be rapidly reversed. In our experiment we have used 90 μg/24 hours in the Swiss mice that weighs about 40 g. In humans the therapeutic dose is 3.5 μg/kg/hour or 6 mg/24 h for a 75 kg man. A comparison of these values tells us that we are working with about 100 times higher values in mice as compared to that used in humans.

### Pharmacokinetic profile of somatostatin in outbred mice

**Figure 2:**
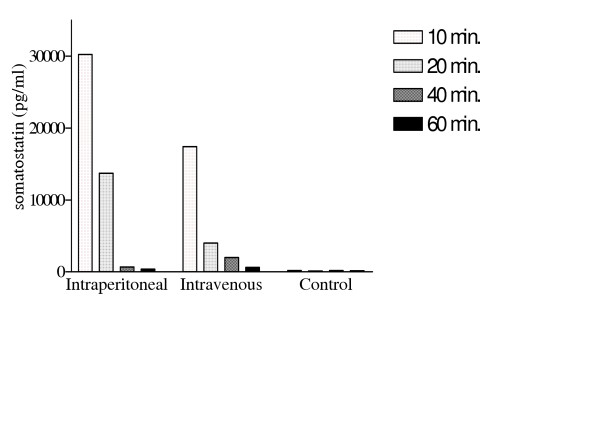
Pharmacokinetic profile of somatostatin in Swiss mice. Figure depicts the breakdown of somatostatin in vivo after intraperitoneal and intravenous administrations. Values were noted after 10, 20, 40 and 60 minutes and compared to a control situation where no somatostatin was administered.

Following an intravenous infusion of a therapeutic dose of somatostatin (250 μg/hour or 6 mg/24 hours) to healthy volunteers, the plasma profile demonstrated that the drug reaches a plateau of 300–3000 pg/ml within 15 minutes. Somatostatin has a very short half-life of 1–3 minutes in man. In animal studies the same profile has been observed in dogs, whereas in rats somatostatin is stable for up to 30 minutes in whole blood, indicating that it is broken down in tissues [17]. To determine the pharmacokinetic profile in outbred Swiss mice, 25 μg of somatostatin was administered per mouse via the intraperitoneal or intravenous route. To assess the break down in these mice, plasma was collected at regular time intervals and circulating somatostatin levels assayed (Figure 2). At 10 minutes post injection somatostatin levels fall to 30233 pg/ml (IP) and 17413 pg/ml (IV). After intravenous injection thus somatostatin appears to be more rapidly degraded than after intraperitoneal injection, however after one hour of injection using either means the levels of circulating somatostatin in blood reaches baseline control levels. Thus in our experimental set up of somatostatin treatment in *S. mansoni *infected mice the schedule of 3 treatments per day (morning, noon and evening), assured that for at least 3 hours per day there were significantly high levels of circulating somatostatin *in vivo*.

### Body and Organ weight after somatostatin treatment

**Figure 3:**
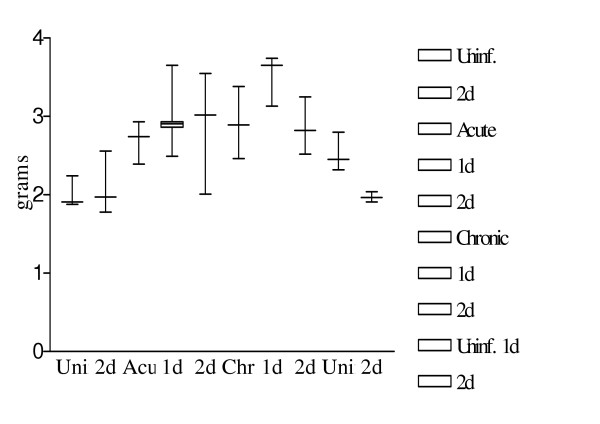
Variations in liver weight after infection and somatostatin treatment. Figure shows the variations in mice liver weights after *S. mansoni *infection and/or somatostatin treatment. Results are depicted as a box and whiskers plot starting from the left – uninfected mice, uninfected mice with 2 days treatment, 10 weeks infected mice, 10 weeks infected mice with 1 day treatment, 10 weeks infected mice with 2 days treatment, 14 weeks infected mice, 14 weeks infected mice with 1 day treatment, 14 weeks infected mice with 2 days treatment. Values represent the 5^th^, 25^th^, 50^th^, 75^th ^and 95^th ^percentiles.

The weight of the liver increased with infection, with a significant rise at the acute (p = 0.01) and chronic stage (p = 0.01) as compared to the uninfected animals (Fig. 3.). Acute and chronic infected animals after 2 days of somatostatin treatment showed no significant change in their liver weights (p = 0.19; 0.90 respectively).

**Figure 4:**
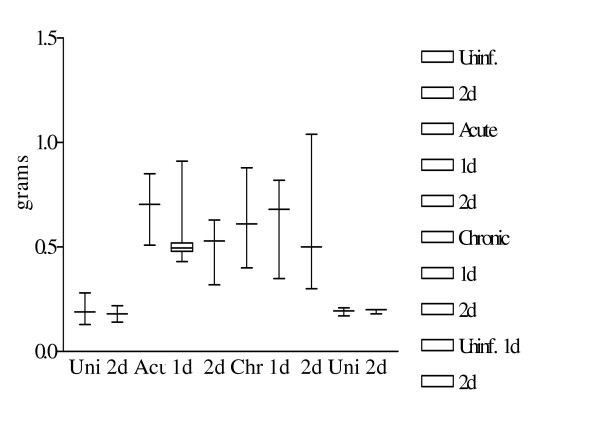
Variations in spleen weight after infection and somatostatin treatment. Figure shows the variations in spleen weight after *S. mansoni *infection and/or somatostatin treatment. Results are depicted as a box and whiskers plot as mentioned in Fig. 3.

Mean spleen weights were significantly increased in acute (p = 0.01) and chronic (p = 0.01) infected animals as compared to uninfected controls (Fig. 4.). Acute and chronic infected animals after 2 days somatostatin treatment showed no significant changes in their spleen weights (p = 0.28; 0.73 respectively).

**Figure 5:**
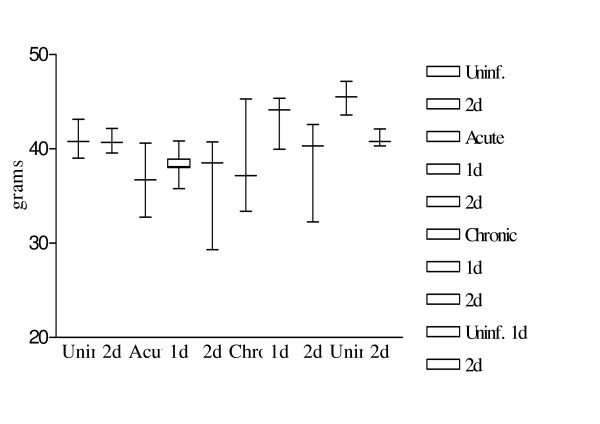
Variations in total body weight after infection and somatostatin treatment. Figure shows the variations in total body weight after *S. mansoni *infection and/or somatostatin treatment. Results are depicted as a box and whiskers plot as mentioned in Fig. 3.

The total body weight of the acute infected animals showed a decrease as compared to uninfected animals however this difference was borderline significant (p = 0.06) (Fig. 5). There were no significant changes in body weight in acute or chronic infected animals after somatostatin treatment.

### Parasite egg count after somatostatin treatment, hepatocyte, granuloma and fibrosis status

**Figure 6:**
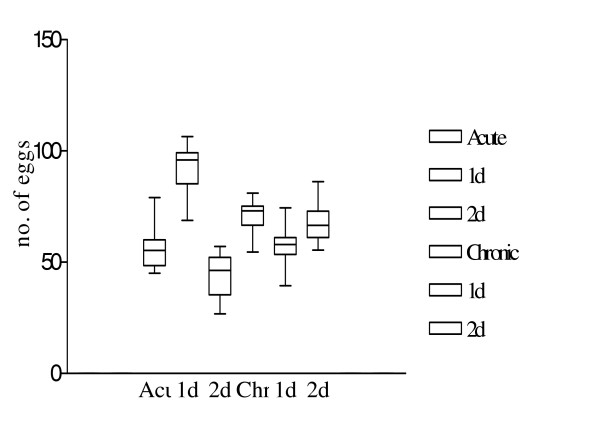
Variations in parasite egg count after somatostatin treatment. Figure shows the variations in parasite egg count after somatostatin treatment. Box and whiskers plot depicting the 5^th^, 25^th^, 50^th^, 75^th ^and 95^th ^percentiles represent egg counts at 10 weeks of infection, variations after 1 day of treatment or 2 days of treatment, egg counts at 14 weeks of infection, variations after 1 day or 2 days of treatment.

*S. mansoni *egg stage parasites were counted in liver sections before and after somatostatin treatment. A series of 10 slides were examined from each sub group, the sections obtained at intervals of at least 40 μm so as to avoid counting repetitive eggs. Two days of somatostatin treatment in acute infected animals significantly reduced the mean parasite egg counts form 56.01 ± 3.34 to 43.76 ± 3.56 (p = 0.03). At the chronic stage of infection, mean egg counts were only significantly (p = 0.006) reduced after 1 day of somatostatin treatment from69.82 ± 2.77 to 56.62 ± 3.02 (Fig. 6.).

Haematoxylin-eosin staining of liver sections displayed the evolution of granuloma formation in infected animals as compared to treated animals. After treatment with somatostatin hepatocyte status remained unaltered, granulomas were not remarkably changed in size or cellularity (results not shown).

### Hydroxyproline determination

**Figure 7:**
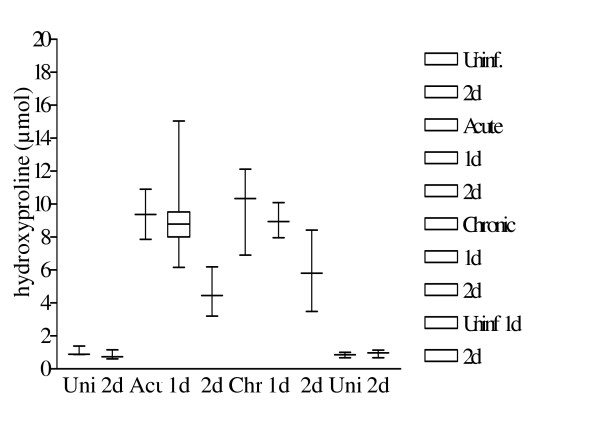
Hydroxyproline levels after somatostatin treatment. Figure depicts the hydroxyproline levels after *S. mansoni *infection and/or somatostatin treatment. Values are represented as a box and whiskers plot as described in Fig. 3.

Hepatic fibrosis is the process of excessive deposition of collagen in the liver. Collagen generation involves 3 polypeptide chains, each chain composed of 19 amino acids consisting of glycine (30%), proline (12%) and two other rather uncommon amino acids – hydroxyproline (10%) and to an even lesser extent hydroxylysine. Proline and hydroxyproline are responsible for the angle (kink) in the polypeptide backbone furthermore hydroxyproline stabilizes the collagen via intramolecular hydroxyl bridges. Normal hydroxylated collagen is equivalent to 10% hydroxyproline in weight. Thus 1 μmol hydroxyproline (MW = 131.13) is equivalent to 1.3113 mg collagen. Hydroxyproline concentrations in the livers (controls versus acute, chronic, treated and untreated) of the Swiss mice are depicted in Table 2 and figure 7. The differences in hydroxyproline concentrations between uninfected and infected mice with or without treatment are illustrated by non-parametric (Mann-Whitney U) or parametric (unpaired Student's t) test as shown in Table 2.

**Table 2:** The hydroxyproline values calculated in the different groups.

	Group 1	Group 2	Group 1 vs. Group 2
No SOM treatment	Uninf.: 1.06 ± 0.10 Acute: 9.37 ± 0.63 p-value: 0.01**	Chronic: 9.65 ± 0.96	Uninf.: 1.06 ± 0.10 Chronic: 9.65 ± 0.96 p-value: 0.007** Acute: 9.37 ± 0.63 Chronic: 9.65 ± 0.96 p-value: 0.90 (NS)

1 day SOM treatment	Acute: 9.37 ± 1.24	Uninf.: 0.85 ± 0.08 Chronic: 8.99 ± 0.61 p-value: <0.0001**	Acute: 9.37 ± 1.24 Chronic: 8.99 ± 0.61 p-value: 0.90 (NS)

2 day SOM treatment	Uninf.: 0.81 ± 0.09 Acute: 4.76 ± 0.58 p-value: 0.007** Acute (untreated): 9.37 ± 0.63 Acute (2d SOM): 4.76 ± 0.58 p-value: 0.01**	Uninf.: 0.93 ± 0.09 Chronic: 5.88 ± 1.13 p-value: 0.02* Chronic (untreated): 9.65 ± 0.96 Chronic (2d SOM): 5.8 ± 1.13 p-value: 0.03*	Uninf. (17 wks.): 0.81 ± 0.09 Uninf. (21 wks.): 0.93 ± 0.09 p-value: 0.41 (NS) Acute: 4.76 ± 0.58 Chronic: 5.88 ± 1.13 p-value: 0.41 (NS)

SOM: somatostatin; Uninf.: uninfected; Inf.: infected; Wks.: weeks;

*Significant difference (p < 0.05); ** significant difference (p < 0.01);

NS: not significant difference

At 10 weeks post infection with 100 *S. mansoni *cercariae, levels of hydroxyproline were significantly higher as compared to that assayed in uninfected animals (p = 0.01) (Fig. 7.). After treatment with somatostatin for 2 days, circulating levels of this collagen at one-week post treatment time were significantly lower as compared to infected animals that were not treated (p = 0.01).

Progression of the infection from week 10 to week 14 did not result in a significant rise in hydroxyproline levels. Treatment of chronically infected animals with a two-day regimen of exogenous somatostatin resulted in a drop in hydroxyproline levels at one-week post treatment. This fall was significant (p = 0.03) as compared to chronic untreated animals.

### Somatostatin levels

**Figure 8:**
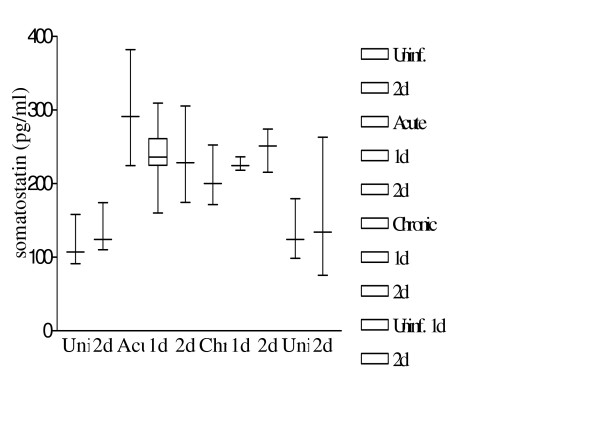
Somatostatin levels after infection and therapy. Figure shows a box and whiskers plot depicting somatostatin values after *S. mansoni *infection and/or somatostatin therapy. Results are described as in Fig. 3.

Somatostatin concentrations in the plasma (controls versus acute, chronic, treated and untreated) of the Swiss mice are depicted in Table 3 and figure 8. The differences in somatostatin concentrations between uninfected and infected mice with or without treatment were illustrated by a non-parametric (Mann-Whitney U) test as shown in Table 3. The distribution of somatostatin levels in all groups is depicted in Figure 8. At 10 weeks post infection with 100 *S. mansoni *cercariae, levels of circulating somatostatin were significantly higher as compared to that assayed in uninfected animals (p = 0.01). After treatment with somatostatin for 1 or 2 days, circulating levels of this neuropeptide at one week post treatment time were not significantly different as compared to infected animals that were not treated.

**Table 3:** The somatostatin levels calculated in the different groups.

	Group 1	Group 2	Group 1 vs. Group 2
No SOM treatment	Uninf.: 119.4 ± 11.99 Acute: 297.3 ± 37.24 p-value: 0.01**	Chronic: 206.1 ± 13.30	Uninf.: 119.4 ± 11.99 Chronic: 206.1 ± 13.30 p-value: 0.008** Acute: 297.3 ± 37.24 Chronic: 206.1 ± 13.30 **p-value: 0.06 (BL)**

1 day SOM treatment	Acute: 240.3 ± 21.30	Uninf.: 131.6 ± 19.34 Chronic: 226.3 ± 5.41 p-value: 0.01**	Acute: 240.3 ± 21.30 Chronic: 226.3 ± 5.41 p-value: 0.71 (NS)

2 day SOM treatment	Uninf.: 136.9 ± 11.69 Acute: 234.2 ± 23.65 p-value: 0.008** Acute (untreated): 297.3 ± 37.24 Acute (2d SOM): 234.2 ± 23.65 p-value: 0.28	Uninf.: 151.6 ± 41.45 Chronic: 247.9 ± 13.11 p-value: 0.11 (NS) Chronic (untreated): 206.1 ± 13.30 Chronic (2d SOM): 247.9 ± 13.11 **p-value: 0.06 (BL)**	Uninf. (17 wks.): 136.9 ± 11.69 Uninf. (21 wks.): 151.6 ± 41.45 p-value: 0.9 (NS) Acute: 234.2 ± 23.65 Chronic: 247.9 ± 13.11 p-value: 0.73 (NS)

SOM: somatostatin; Uninf.: uninfected; Inf.: infected; Wks.: weeks;

* Significant difference (p < 0.05); ** significant difference (p < 0.01); BL: borderline significant difference; NS: not significant difference

Progression of the infection from acute (week 10) to chronic (week 14) stages resulted in a drop in the somatostatin levels. Comparison of somatostatin levels between these two time points depicted borderline significant differences (p = 0.06). Treatment of chronically but not of acute infected animals with a two-day regimen of exogenous somatostatin resulted in an elevated level of circulating hormone at one-week post treatment. This rise in circulating somatostatin level was borderline significant (p = 0.06) as compared to chronic untreated animals.

## Discussion and conclusions

The neuropeptide somatostatin is one of the major regulatory hormones in the central nervous system and the digestive tract. Improved knowledge of the patho-physiological processes of schistosomiasis can be obtained by studying the regulatory mechanisms of somatostatin in the human body. The activity of somatostatin is mediated via binding to specific cell surface receptors. To understand the expression, regulation, true role and distribution of these receptors, animal models come in helpful in defining the physiological and pathological conditions set up by schistosomiasis. Somatostatin, produced by neuroendocrine, inflammatory and immune cells inhibits various cellular functions including secretions, motility, and proliferation [18,13]. Preprosomatostatin (pp-SOM), a precursor of somatostatin is synthesized in the pancreas, the gastrointestinal network, and the brain. Two products of this pp-SOM are somatostatin-14, a 14 amino acid peptide, and somatostatin-28 (SS-28) that contain the SS-14 but is prolonged at the N-terminus. Its action is mediated by five specific somatostatin receptors (SSTR1 – SSTR5), which belong to the G protein-coupled receptor family.

The last years we have been studying the potential of somatostatin in modulating the pathology caused by schistosomiasis, in particular hepatic fibrosis. The trematode, *Schistosoma mansoni *is phylogenetically the oldest class of invertebrate in which we have identified immunopositivity to the somatostatin receptors SSTR2 and SSTR3 (manuscript submitted). The presence of these receptors on the parasite stages indicate that circulating somatostatin levels might interact with the pathogenic parasitic stages and hence influence hepatic fibrosis caused by this parasite.

The helminth parasite *Schistosoma mansoni *and its related species cause the fibrotic hepatic disease, schistosomiasis. Fibrosis is the process of excessive deposition of collagen in a tissue together with other extracellular matrix (ECM) components like fibronectin, proteoglycans, laminin and elastin. To some extent ECM deposition is necessary for wound healing since it provides strength and temporary structure to damaged tissues. However, if not limited this process is pathogenic. Liver fibrosis can be particularly detrimental leading to portal hypertension and its attendant sequelae that include splenomegaly and rupture-prone gastroesophageal varices.

The collagen peptide-synthesis in the liver of infected mice attains a peak activity at 8 weeks post infection. The triple polypeptide chains, assembled to form procollagen, once leaving the cell come together to form polymeric collagen fibers. The hydroxyproline residues take care of the stability of the three dimensional helix via the formation of hydroxyl bridges between the polypeptide chains. The important points in collagen biosynthesis are thus the positioning of the hydroxylproline residues in the triple backbone structure, the knipping off of the propeptides from procollagen to form collagen as it leaves the cell and finally the cross-linking to form fibers. The deposition of fibronectin and heparansulphate round the egg rises rapidly till week 11 post infection after which levels plateau off. All these extracellular matrix components interact with different cells and modulate various cellular activities like migration, proliferation, differentiation, chemotaxis etc. [19].

Somatostatin that has earned itself the nickname 'endocrine cyanide' due to its slowing down a number of biological processes in the body, might add another feature to its ubiquitous nature – that of reducing fibrosis in *Schistosoma mansoni *infections.

We have quantified fibrosis (amount of collagen) generated by *S. mansoni *infections and the reductions after somatostatin treatments by measuring the levels of hydroxyproline at each time point.

In acute and chronic infected animals that were untreated, the increased collagen levels were associated with increased somatostatin levels respectively. Two days treatment of acute and chronic infected animals with somatostatin significantly reduced hydroxyproline levels. Endogenous somatostatin levels were not affected during the acute phase after somatostatin treatment although in chronic infected mice endogenous somatostatin levels tended to increase. However, at both time points, a significant reduction in parasite egg counts was observed, suggesting that therapeutic doses of somatostatin might, by binding to somatostatin receptors on the parasite surface, inhibit the production of the parasite stage that induces the inflammatory granulomatous response that can lead to fibrosis.

Reports by Reynaert *et al*. [12] have identified the somatostatin receptors SSTR1, 2 and 3 on the hepatic stellate cells that are responsible for collagen synthesis. In our experiments exogenous somatostatin might also have bound to these receptors thereby inhibiting collagen synthesis.

Taken together we have elucidated the therapeutic capacity of somatostatin in reducing hepatic fibrosis. This report in fact confirms a previous report by Mansy [20], where using a somatostatin synthetic analog octreotide in *S. mansoni *infected animals, a reduction in hepatic fibrosis was noted. Since octreotide is limited in binding to somatostatin receptors, our results with natural somatostatin that binds to all 5 somatostatin receptors equally well assures us of the anti fibrotic capacities of this neuropeptide hormone, and points to its potential use in human conditions.

## Competing interests

The author(s) declare that they have no competing interests.

## Authors' contributions

SC designed this study, performed the parasite egg counting, and statistical analysis. GV carried out the animal infection, somatostatin treatment, and hydroxyproline determinations. IDP and TP performed the somatostatin detection in the mice plasma. EVM participated in the design of the study and coordination. All authors read and approved the final manuscript.

## Pre-publication history

The pre-publication history for this paper can be accessed here:


